# The relationship between work–family conflict and occupational burnout among nurses: the mediating role of career calling

**DOI:** 10.3389/fpubh.2026.1740207

**Published:** 2026-04-08

**Authors:** Zongting Luo, Jian Zhou, Lifang Chen, Yan Zhang, Min Wang, Xiaolin Ma, Zehua Li, Lan Tao, Lijuan Chen, Yue Chang

**Affiliations:** 1 Department of Nursing, The Third People’s Hospital of Chengdu, Chengdu, China; 2 Department of Orthopedics, The First Affiliated Hospital of Chengdu Medical College, Chengdu, China; 3 Department of Cardiology, The Third People’s Hospital of Chengdu, Chengdu, China; 4 Department of Nursing, Pengzhou People’s Hospital, Chengdu, China; 5 School of Nursing, Chengdu University, Chengdu, China; 6 Dean’s Office, Chengdu Seventh People’s Hospital, Chengdu, China

**Keywords:** career calling, cross-sectional study, mediating role, nursing research, occupational burnout, work–family conflict

## Abstract

**Background:**

Occupational burnout has an important impact on the physical and mental health of nurses, stability of the nursing team, and patient safety. As significant contributors to the effective operation of medical and health institutions, clinical nurses experience high occupational burnout because of the comprehensive effects of many factors, such as family and work pressures. Therefore, it is necessary to fully understand the factors associated with nurses’ occupational burnout and their interrelationships, to support the development of targeted interventions to reduce occupational burnout among clinical nurses.

**Objective:**

To explore the relationship between work–family conflict, career calling, and occupational burnout among nurses. In addition, we studied whether career calling plays a mediating role between work–family conflict and occupational burnout.

**Methods:**

A cross-sectional survey was conducted with 1,145 nurses. Data were collected using a general information questionnaire, Work-Family Behavioral Role Conflict Scale, Career Calling Scale, and Maslach Burnout Inventory. Structural equation models were used to build the model. A STROBE checklist was used to report the results.

**Results:**

Nurses’ occupational burnout was positively related to work–family conflict but negatively correlated with career calling. Meanwhile, career calling mediates the relationship between work–family conflict and occupational burnout.

**Conclusion:**

Nurses’ career calling plays a mediating role in the relationship between work–family conflict and occupational burnout. The findings of this study can provide essential information to nursing managers to formulate targeted strategies to reduce occupational burnout among nurses.

## 1 Introduction

Occupational burnout refers to physical and mental exhaustion caused by long-term high-intensity and high-load work, mainly emotional exhaustion, depersonalization tendencies, and a low sense of accomplishment (1). With an aging population and increasing health needs of patients, the workload of nurses continues to increase (2). However, there is a relative shortage of nursing human resources (3, 4), and nurses have a high incidence of occupational burnout because of their heavy workload, high work risk, irregular work and rest, and other peculiarities of their occupational nature (5, 6). Studies have demonstrated that nurses’ occupational burnout not only affects their physical and mental health and quality of life (7), leading to an increase in turnover (8, 9), but also reduces the quality of nursing services and jeopardizes patient safety (10). Therefore, it is of great practical significance to explore the influencing factors and mechanisms of nurses’ occupational burnout.

Work–family conflict is a role conflict that occurs in individuals when the demands of the work and family spheres cannot be balanced (11). A study by Yarifard et al. including 256 nurses from northwestern Iran found that work–family conflict had a negative effect on occupational burnout (12); but the intrinsic action path of this effect still needs to be further clarified. From the perspective of Conservation of Resources (COR) theory (13), individual core resources such as time, emotion and energy are limited. The essence of work–family conflict is the competition and consumption process of scarce resources between job role requirements and family role responsibilities. This competition for resources will lead to the loss of individual resources faster than the replacement rate, forming a “resource deficit,” and occupational burnout is the typical stress response caused by resource exhaustion, which provides the core theoretical support for the relationship between work–family conflict and occupational burnout.

Career calling is an attribute of strong passion within an individual for a field with positive emotions (14). According to the COR theory, career calling belongs to high-value internal psychological resources (15), which is self-regenerative—individuals with a higher sense of career calling can perceive value and meaning from work, generate continuous internal motivation (16), and then buffer the negative impact caused by the depletion of external resources. This self-regenerative feature enables career calling to act as an intermediary that connects work–family conflict (a resource-consuming stressors) and occupational burnout (a resource exhaustion outcome), forming a complete resource conservation path. From the perspective of the specific mechanism of resource consumption and regeneration, the essence of work–family conflict is the competition and consumption process of individual scarce resources by work roles and family roles: Nurses need to invest a lot of time and energy in high-intensity clinical work, and also take care of parents, children and other family responsibilities. This dual role demand will lead to resource competition. If more resources are put into work, they will not be able to meet the needs of family role, which will cause family guilt, and then consume emotional resources. If more resources are invested in the family, it will affect the completion of work tasks, reduce the sense of achievement, and also lead to the loss of resources. This constant resource consumption gradually erodes the individual’s internal psychological resources, including a sense of career calling: When nurses are plagued by the dual pressure of work and family for a long time and cannot get enough value feedback from work, their enthusiasm and identity for the nursing profession will gradually decrease, and the level of career calling will decrease, that is, the work–family conflict will indirectly lead to the loss of internal psychological resources (career calling) through the consumption of external resources. As an internal psychological resource, the decline in the level of career calling will further weaken the individual’s ability to regenerate resources. Without the support of career calling, it is difficult for nurses to obtain a sense of value and motivation from work, and they cannot replenish the consumed resources through self-motivation, and then fall into a vicious circle of “resource depletion—decreased sense of mission—aggravated burnout.” This eventually leads to an increase in the level of occupational burnout. Studies have confirmed that career calling can reduce the level of occupational burnout by improving work engagement and alleviating negative emotions (16). Other studies have pointed out that family-supportive supervision behaviors can help employees balance work and life by reducing work–family conflicts, so as to achieve career calling (17), which initially reveals the close relationship between work–family conflicts and career calling, suggesting that career calling may play an important mediating role between them.

Clinical nurses are important members of the effective functioning of healthcare organizations; however, the combined effects of multiple factors, such as family and work pressures, have caused nurses to face higher levels of occupational burnout (18). At present, there are no systematic studies on the relationship between work–family conflict, career calling and occupational burnout among clinical nurses in Sichuan Province, and the action path between variables remains unclear. Although similar mediation models have been reported in related occupational contexts, this study focuses on clinical nurses in Sichuan Province and is a contextual extension of the existing research. As a classical framework to explain the relationship among stressors, psychological resources, and negative outcomes, COR theory provides a solid theoretical basis for the construction of the relationship among the three. As a resource-consuming stressors, work–family conflict may not only be directly related to occupational burnout, but also may be indirectly related to occupational burnout by depleting the internal psychological resource of career calling. Career calling, as a key psychological resource, may be negatively correlated with occupational burnout by buffering resource depletion and improving the sense of meaning in work. Based on the above theoretical analysis and literature review, this study puts forward the following research hypotheses:

*H1*: Nurses’ work–family conflict is positively correlated with occupational burnout.

*H2*: Nurses’ work–family conflict is negatively correlated with career calling.

*H3*: Nurses’ career calling is negatively correlated with occupational burnout.

*H4*: Nurses’ career calling plays a mediating role between work–family conflict and occupational burnout.

Guided by the COR theory, this study focuses on the group of clinical nurses in Sichuan Province. By constructing a structural equation model, it explores the current situation of nurses’ work–family conflict, career calling and occupational burnout, verifies the above research hypotheses, clarifies the mediating role of career calling, provides a basis for nursing managers to formulate targeted strategies to reduce occupational burnout among clinical nurses.

## 2 Methods

### 2.1 Study design

This study used a descriptive cross-sectional design to examine the relationship between work–family conflict, career calling, and occupational burnout among nurses. Whether career calling mediates work–family conflict and occupational burnout. The Strengthening the Reporting of Observational Studies in Epidemiology (STROBE) checklist (19) was used to ensure research quality.

### 2.2 Participants

This study was conducted from October 2024 to November 2024. Convenience sampling method was used to select clinical nurses from 41 tertiary hospitals in Sichuan Province as the study subjects. The inclusion criteria were as follows: (a) working registered nurse; (b) at least 1 year of clinical nursing experience; and (c) informed consent and voluntary participation in this study. The exclusion criteria was absence from work for more than 1 month owing to vacation, study away, or other reasons.

The sample size was calculated using the method introduced by Jin et al. (20), with an invalid questionnaire rate of 15%. Therefore, at least 368 participants were included in our study.

### 2.3 Data collection instruments

#### 2.3.1 General information questionnaire

The authors designed a general information questionnaire to collect demographic characteristics of clinical nurses, including sex, age, education level, marital status, years of work experience, professional title, average number of night shifts per month, average number of hours of overtime work per week, attitudes toward the nursing profession, and job compensation match.

#### 2.3.2 Work-family behavioral role conflict scale

The scale was developed by Clark et al. (21) in 2019 and sinicized by Sun et al. (22) in 2023 to measure the level of individual work-family behavioral role conflict. It consists of two dimensions, work-to-family and family-to-work, with 30 items. The Likert 5-level scoring method was used, with never, seldom, sometimes, often, and very often scored from 1 to 5, and the total score ranged from 30 to 150; the higher the score, the higher the level of work–family conflict among nurses. The Cronbach’s alpha coefficient of the original scale was 0.958, and 0.973 in this study.

#### 2.3.3 Career calling scale

The Career Calling Scale developed by Dobrow et al. (23) in 2011, “Chinese-eized” by Pei et al. (24) in 2015, and revised by Shen et al. (25) in 2022 was used to evaluate nurses’ career calling levels. It is a one-dimensional scale containing 12 items. The Likert 5-level scoring method was used, with “Strongly Disagree” scoring 1 and “Strongly Agree” scoring 5; the higher the score, the stronger the nurses’ sense of career calling. The Cronbach’s alpha coefficient of the original scale was 0.940, and 0.954 in this study.

#### 2.3.4 Maslach burnout inventory

Nurses’ occupational burnout level was assessed using the Maslach Burnout Inventory. This scale was developed by Maslach and Jackson (26) and translated by Feng et al. (27). The scale consists of 22 items, including three dimensions: emotional exhaustion (9 items), depersonalization (5 items), and personal accomplishment (8 items). The Likert 7-level scoring method was used, with scores ranging from 0 to 6, from “never” to “every day,” and the personal accomplishment dimension was scored inversely, with a total score ranging from 0 to 132, with higher scores indicating a higher degree of occupational burnout. Individuals with an emotional exhaustion score of ≥27, a depersonalization score of ≥10, and a personal accomplishment score of ≥15 are in a state of burnout in the respective dimension. If none of the three dimensions were in a burnout state, it was considered zero burnout; if a single dimension was in a burnout state, it was considered mild burnout; if two dimensions were in a burnout state, it was considered moderate burnout; and if all three dimensions were in a burnout state, it was considered severe burnout. The Cronbach’s alpha coefficient of the original scale was 0.834, and 0.967 in this study.

### 2.4 Ethical considerations

The study was performed in accordance with the Declaration of Helsinki and approved by the Ethics Committee of the Third People’s Hospital of Chengdu (Number: 2024-S-263; date: 22 August 2024). Informed consent was obtained from all participants electronically before the study. After the introduction of the study purpose, the nurses were asked whether they would like to participate in the survey. The official survey interface would be entered after clicking “Agree to participate.” Participants were informed that they could withdraw from the study at any time. All data were confidentially treated.

### 2.5 Data collection

Before the formal study, we randomly selected an appropriate number of people with the same demographic characteristics as the participants for pre-survey, which showed that the data collection instruments had good face validity and content reliability. During the formal survey, data were collected through an online questionnaire platform. After obtaining consent from the person in charge of the hospital, with the help of the nursing department and head nurse, the QR code was sent to the nurses’ workgroup, and the nurses scanned the QR code to complete the questionnaire anonymously online. To reduce potential common method bias, the first page of the questionnaire uses unified guidance to explain the research purpose, significance, and questionnaire filling method, and emphasizes the anonymity and confidentiality of the questionnaire. Each IP address can only be filled in once, and all questions are set as ‘required’. Only after all the questions are completed can they be submitted successfully, which may reduce careless responding and evaluation apprehension. After completing the survey, two researchers checked the collected questionnaires and excluded those with a clear regularity or uniformity. A total of 1,213 questionnaires were collected, of which 1,145 were valid, with an effective rate of 94.39%.

### 2.6 Data analysis

SPSS software (version 26.0) was used for statistical analysis of the data. General data are expressed as frequencies and component ratios (%). The mean ± standard deviation was used for the work–family conflict, career calling, and occupational burnout scores. Pearson coefficient was used to test the correlation among the three variables, and the significance level was *p* < 0.05. To explore the relationship between work–family conflict, career calling, and occupational burnout in nurses and the mediating role of career calling in these relationships, a structural equation model was established using SPSS Amos 28.0 software. The mediation hypothesis was tested using the bootstrap method, with the sampling number set at 5000 replications. Statistical significance was defined as a deviation-corrected percentile bootstrap with a 95% confidence interval (CI) that did not contain a 0. In AMOS, we first assessed the measurement model using confirmatory factor analysis (CFA) prior to estimating structural paths. Work–family conflict was specified as a latent construct indicated by two subscale scores (work-to-family and family-to-work), and occupational burnout was specified as a latent construct indicated by emotional exhaustion, depersonalization, and personal accomplishment. For greater measurement-model transparency, career calling was additionally assessed in the CFA as a latent construct indicated by the 12 items of the Career Calling Scale. Modification indices were inspected after model estimation, but no post-hoc model modifications were applied and no correlated error terms were added. To assess potential common method bias, Harman’s single-factor test was performed using an unrotated exploratory factor analysis including all questionnaire items, and a one-factor CFA model (all observed indicators loading onto a single latent factor) was additionally estimated and its model fit evaluated to determine whether a single common factor could account for the covariance among the measures.

## 3 Results

### 3.1 Characteristics of the participant

Of the 1,145 nurses included in the analysis, 95.9% were female and 7.42% had senior titles. The demographic characteristics of the nurses who participated in this study are illustrated in Table 1.

**Table 1:** The demographic characteristics of participants (*N* = 1,145).

Variables	Items	*N*	%
Sex	Male	47	4.10
	Female	1,098	95.90
Age	20–30	456	39.83
	31–40	498	43.49
	>40	191	16.68
Education level	Secondary vocational	10	0.87
	Junior college	319	27.86
	Undergraduate	816	71.27
Marital status	Married	842	73.54
	Unmarried	267	23.32
	Divorced/widowed	36	3.14
Years of work experience	<5	221	19.30
	5–10	362	31.62
	11–20	410	35.81
	>20	152	13.27
Professional title	Junior	558	48.74
	Intermediate	502	43.84
	Senior	85	7.42
Average number of night shifts per month	<4	536	46.81
	4–8	463	40.44
	>8	146	12.75
Average number of hours of overtime work per week	<5	818	71.44
	5–10	248	21.66
	>10	79	6.90
Attitudes toward the nursing profession	Like	732	63.93
	Does not matter	328	28.65
	Dislike	85	7.42
Job compensation match	Perfect match	195	17.03
	Basic match	685	59.83
	Complete mismatch	265	23.14

### 3.2 Descriptive statistics of work–family conflict, career calling, and occupational burnout by nurses

The means and standard deviations of the total and dimensional scores for each study variable are listed in Table 2. The mean scores for work–family conflict, career calling, and occupational burnout were 78.69 (SD = 28.19), the mean score for career calling was 40.47 (SD = 11.90), and the mean score for occupational burnout was 46.81 (SD = 26.99), respectively.

**Table 2:** Scores of work–family conflict, career calling, and occupational burnout.

Variables	Mean	SD	Min	Max
Work–family conflict	78.69	28.19	31	150
Work-to-family	39.66	15.76	15	75
Family-to-work	39.03	15.60	15	75
Career calling	40.47	11.90	18	60
Occupational burnout	46.81	26.99	1	132
Emotional exhaustion	18.98	11.45	0	54
Depersonalization	10.62	6.35	0	30
Personal accomplishment	17.20	9.98	0	48

### 3.3 Correlation analysis of work–family conflict, career calling, and occupational burnout by nurses

Pearson’s statistical results demonstrated that work–family conflict was negatively correlated with career calling (*r* = −0.354, *p* < 0.01) but positively correlated with occupational burnout (*r* = 0.413, *p* < 0.01). In addition, career calling was negatively correlated with occupational burnout (*r* = −0.394, *p* < 0.01) (Table 3).

**Table 3:** Correlations of work–family conflict, career calling, and occupational burnout.

Variables	1	2	3
1. Work–family conflict	1		
2. Career calling	−0.354^**^	1	
3. Occupational burnout	0.413^**^	−0.394^**^	1

**p < 0.01.

### 3.4 The mediating role of career calling on work–family conflict and occupational burnout

**Figure 1:**
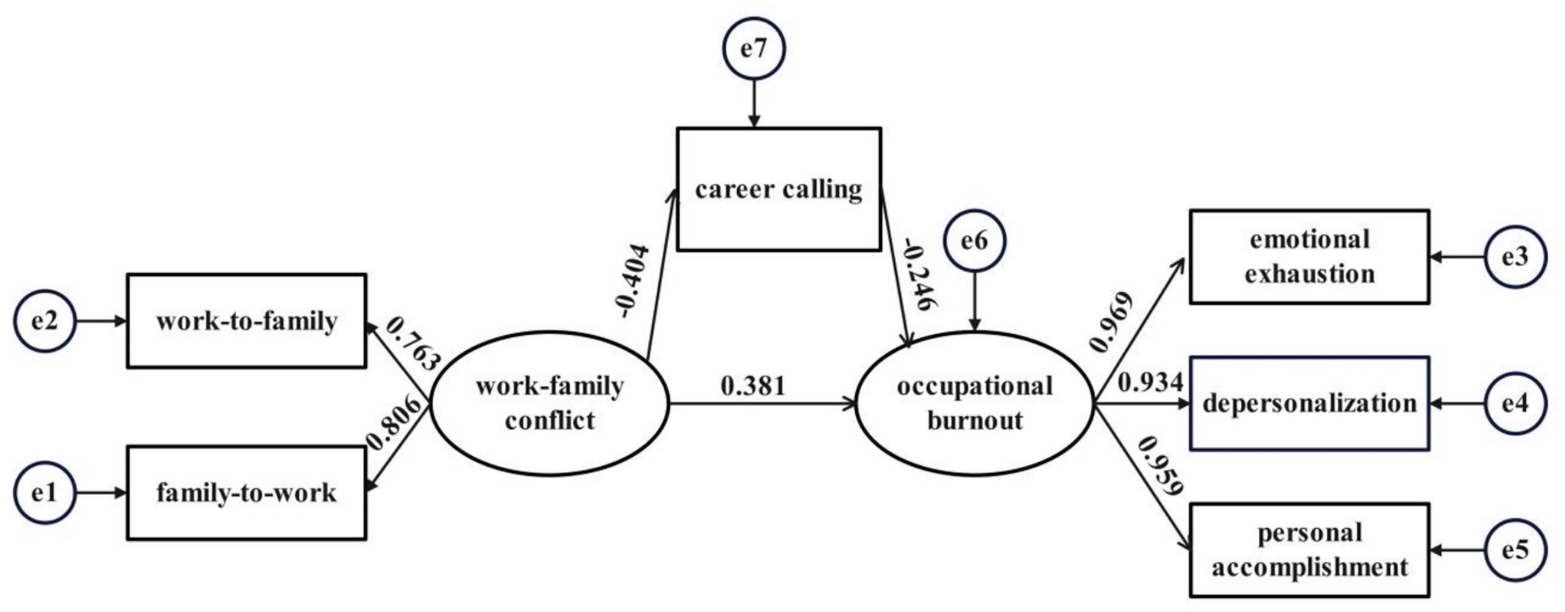
Standardized parameter estimates for the final structure model.

Before testing the mediation paths, we assessed the measurement model using CFA in AMOS. The measurement model showed good fit to the data (χ^2^ = 173.226, df = 116, *p* < 0.001; χ^2^/df = 1.493; RMSEA = 0.021, PCLOSE = 1.000; CFI = 0.996; TLI = 0.996; GFI = 0.982; AGFI = 0.976). Modification indices were inspected after model estimation, but no post-hoc model modifications were applied and no correlated error terms were added. To assess potential common method bias, Harman’s single-factor test showed that the first factor accounted for 36.634% of the total variance, suggesting that a single general factor did not explain the majority of covariance among the measures. In addition, the one-factor CFA model, in which all indicators loaded onto a single latent factor, demonstrated poor fit (χ^2^ = 478.875, df = 9, *p* < 0.001; χ^2^/df = 53.208; RMSEA = 0.214, PCLOSE = 0.000; CFI = 0.913; TLI = 0.854; GFI = 0.884; AGFI = 0.730), indicating that common method bias was unlikely to pose a serious threat to the validity of the study conclusions. The fit indices of the final structural model are presented in Table 4. The relationships between the factors in the fitting model are illustrated in Figure 1. Work–family conflict had a direct negative role on career calling (β = −0.404, *p* < 0.01), and career calling had a direct negative role on occupational burnout (β = −0.246, *p* < 0.01). Furthermore, work–family conflict directly impacted occupational burnout (β = 0.381, *p* < 0.01).

**Table 4:** Structural equation model fitting index.

Model index	χ^2^/df	RMSEA	GFI	NFI	IFI	CFI	RFI	TLI
Recommended value	≤5	≤0.08	≥0.90	≥0.90	≥0.90	≥0.90	≥0.90	≥0.90
Measured value	1.234	0.014	0.997	0.998	1.000	1.000	0.997	0.999

RMSEA, root mean square error of approximation; GFI, goodness of fit index; NFI, normed fit index; IFI, incremental fit index; CFI, comparative fit index; RFI, relative fit index; TLI, Tucker-Lewis index.

Utilizing the bootstrap method 5,000 times to yield a value under 95% CI, the mediating role of career calling on work–family conflict and occupational burnout was further examined. Work–family conflict had an indirect impact of 0.099 on occupational burnout (Table 5). A bootstrap 95% CI of 0.065–0.114 did not include a value of 0, demonstrating the statistical significance of the mediating role.

**Table 5:** The bootstrap analysis results of mediation effect test.

Variables	Standardized effect value	95% CI	
		Lower	Upper
Indirect effect	0.099	0.065	0.114
Direct effect	0.381	0.266	0.406
Total effect	0.480	0.353	0.493
*R*	0.206	0.153	0.280

R = indirect effect/total effect.

## 4 Discussion

This study found that nurses’ occupational burnout was positively related to work–family conflict but negatively correlated with career calling. The findings also support the idea that career calling plays a mediating role between work–family conflict and occupational burnout.

The results of this study demonstrated that nurses’ work–family conflict was positively correlated with occupational burnout (*p* < 0.001), indicating that the higher the level of work–family conflict, the more pronounced their occupational burnout. This is similar to the research conclusion drawn by Bu et al. that nurses’ occupational burnout is a multidimensional and complex phenomenon influenced by multiple factors, such as work environment, family support situation, and individual psychological state (18). Work–family conflict includes two aspects: the impact of work on the family and the impact of family on work, and it occurs when individuals have difficulty balancing the relationship between work and family (28). Nursing work is intense, fast-paced, and performed in shifts around the clock (29, 30). Nurses must take care of family responsibilities, such as caring for parents and raising children, while doing their essential work. If nurses invest too much time and energy in their work, they are unable to meet the requirements of their family roles and functions well, which will make nurses feel guilty about their families, reducing their enthusiasm and motivation for work. Similarly, if excessive family burdens interfere with nurses’ work commitment and the completion of their work-related tasks, it will reduce their sense of personal fulfillment, which will eventually lead to occupational burnout (31). Yang et al. found that work-family support was a protective factor against occupational burnout among primary health workers through a survey of 8,135 primary health workers in 320 primary health care organizations (32), which also confirms the idea that reducing work–family conflict is beneficial for reducing occupational burnout. This suggests that clinical nursing managers should first strengthen the attention to the work–family conflict of nursing staff, such as using the validated work–family conflict scale to regularly assess the level of work–family conflict of nursing staff, so as to identify high-risk groups early and implement targeted interventions. Secondly, on the basis of traditional management methods such as creating a harmonious department atmosphere and organizing regular mental health lectures, they should combine with emerging concepts such as digital management, such as introducing a mobile nursing system, an intelligent scheduling system, and a consumables management system, the work process and scheduling mode were scientifically optimized to improve work efficiency. At the same time, a digital family support platform was built to provide online resources such as parenting knowledge, pension services, and psychological counseling to help nurses effectively solve practical difficulties in family affairs and alleviate the conflict between work and family so as to reduce the occurrence of occupational burnout.

The results of this study demonstrated that nurses’ career calling was negatively correlated with occupational burnout (*p* < 0.001), indicating that the higher the level of nurses’ career calling, the lower their occupational burnout. This is similar to the findings of He et al. that career calling has a mitigating effect on occupational burnout (33). As an intrinsic driving force, career calling can motivate individuals to fully mobilize existing resources and play a stimulating role in effectively coping with stressful environments, clarifying personal goals, and pursuing the realization of self-worth (34). Clinical nurses, who have the most frequent contact with patients in the healthcare system, are exposed to many sources of stress at work, including heavy workloads, risk of infection, handling challenging patients, and strained interpersonal relationships (35). If nurses fail to cope positively with occupational stress, they lose enthusiasm for their work, which increases their risk of occupational burnout. In addition, career achievement recognition and professional development opportunities are inextricably linked to nurses’ occupational burnout (36). If nurses fully recognize their own career values and plan their career paths, it will help enhance work motivation and reduce the occurrence of occupational burnout. Zhao et al. observed that individuals with higher levels of career calling had a lower likelihood of experiencing occupational burnout (37). Career calling can help individuals correctly view challenges and pressures in the workplace and seek support, better regulate their negative emotions and sense of powerlessness at work under pressure, continuously adapt to their professional roles, and maintain their enthusiasm for work. In contrast, individuals with higher levels of career calling will actively search for career values that are in line with their own and will be willing to dedicate their time, energy, and emotions to their work and have clearer work goals, which in turn will have a more positive impact on their work attitudes and behaviors. It is suggested that in managing occupational burnout among clinical nurses, it is important to focus on cultivating career calling. For example, nurses’ professional mission and value training should be included in the training content, and modules such as professional values education, excellent nurse model, and career planning workshop should be set up to stimulate nurses’ sense of nursing mission and professional value; help nurses explore their career interests, aspirations, and strengths to clearly understand their career development paths; and provide philosophical lectures to help nurses correctly face pressure in their work. At the same time, establish a digital career achievement file, systematically record the key information such as nurses’ work performance, training results, and patient satisfaction evaluation, intuitively present the career growth trajectory through data visualization technology, and continuously stimulate the motivation of nurses to pursue a career. The aim of reducing occupational burnout is achieved by improving nurses’ career calling.

This study found that the total effect of work–family conflict in predicting nurses’ occupational burnout was significant, and work–family conflict still predicted nurses’ occupational burnout when career calling was introduced, indicating that work–family conflict not only directly associated with nurses’ occupational burnout but also indirectly associated with occupational burnout through career calling. The indirect effect of work–family conflict on occupational burnout through career calling accounted for 20.6% of the total effect, indicating that career calling is an important way for nurses to reduce occupational burnout through work–family conflict. The reasons for this are as follows: clinical nurses have significant work–family conflict owing to shift work, long working hours, and a high number of night shifts (38). The emergence of work–family conflict will cause emotional labor among nurses. If these emotions persist and gradually interfere with work, they will continue to deplete the individual’s limited psychological and emotional resources. This long-term depletion of emotional resources will further weaken the intrinsic driving force of the work (i.e., reduce the level of their career calling), which will then negatively affect work motivation and work efficiency and ultimately lead to the occurrence of occupational burnout. In contrast, if nurses can balance the relationship between work and family and the family and department provides sufficient support to reduce work–family conflicts, they can have more energy and time devoted to their work and personal development, which will stimulate their enthusiasm and motivation for work, continuously pursue self-worth, and realize their career calling, which is conducive to alleviating occupational burnout. This is consistent with Sharma et al., who suggested that the positive impact of career calling on job performance and subjective career success tends to increase exponentially when employees are supported by colleagues, supervisors, and the organization (39). It is suggested that in the process of alleviating nurses’ occupational burnout, clinical nursing managers should not only pay attention to nurses’ personal work and family life, but also actively help them to coordinate work and family needs, such as optimizing shift arrangements, strictly controlling excessive overtime, and improving family-friendly policies (such as family care flexible leave, child care and pension support services). We should also consider the internal driving force of work, such as clarifying the career promotion path, timely recognizing the professional contribution of nurses, and continuously strengthening their career calling level, so as to effectively prevent or alleviate the level of occupational burnout of clinical nurses.

## 5 Limitations

Owing to the limitations of time and human resources, this study adopted a cross-sectional survey design and could not infer the causal relationship between variables. Although all focal variables were self-reported, the results of Harman’s single-factor test and the poor fit of the one-factor CFA model suggest that common method bias is unlikely to pose a serious threat to the validity of the present findings. At the same time, the study did not include potential moderating variables such as individual characteristics and organizational environment factors, which may lead to some variation not being fully captured and may affect the comprehensiveness of the study results to a certain extent. Longitudinal studies should be conducted in the future to explore trajectory changes in nurses’ occupational burnout.

## 6 Conclusion

In summary, this study focused on a population of clinical nurses and, combined with COR theory, revealed part of the mediating role of career calling between work–family conflict and occupational burnout. The integration of digital innovation into traditional management, from the two dimensions of “reducing resource consumption” and “supplementing psychological resources,” provides a new practical idea for relieving nurses’ occupational burnout. On the one hand, by creating a harmonious atmosphere in the department, carrying out regular mental health lectures, and combining an intelligent scheduling system and a mobile nursing platform to optimize the work process, a digital family support platform was built to help nurses reduce the resource competition between work and family and reduce work–family conflicts from the source. On the other hand, special training can stimulate nurses’ sense of career calling and value identity, assist in planning personalized career development paths, establish digital career achievement files, visualize career growth trajectories, and continuously strengthen the reserve and regeneration ability of the core psychological resource of career calling. The theoretical and practical significance of this study is that the combination of occupational environmental conflict, intrinsic occupational motivation, and occupational mental health enriches the understanding of the influencing factors and mechanisms of nurses’ occupational burnout and provides an empirical basis for the formulation of targeted and operable nursing management intervention programs. However, its long-term practical effect and scene suitability still need to be further explored.

## Data Availability

The raw data supporting the conclusions of this article will be made available by the authors, without undue reservation.
